# Laser Sintering Approaches for Bone Tissue Engineering

**DOI:** 10.3390/polym14122336

**Published:** 2022-06-09

**Authors:** Jeremy N. DiNoro, Naomi C. Paxton, Jacob Skewes, Zhilian Yue, Philip M. Lewis, Robert G. Thompson, Stephen Beirne, Maria A. Woodruff, Gordon G. Wallace

**Affiliations:** 1 ARC Centre of Excellence for Electromaterials Science, Innovation Campus, Intelligent Polymer Research Institute, AIIM Facility, University of Wollongong, Wollongong, NSW 2522, Australia; jdn388@uowmail.edu.au (J.N.D.); zyue@uow.edu.au (Z.Y.); sbeirne@uow.edu.au (S.B.); 2 Australian Research Council Industrial Transformation Training Centre in Additive Biomanufacturing, Brisbane, QLD 4059, Australia; n.paxton@qut.edu.au (N.C.P.); mia.woodruff@qut.edu.au (M.A.W.); 3 Centre for Biomedical Technologies, Queensland University of Technology, Brisbane, QLD 4059, Australia; j.skewes@qut.edu.au; 4 Department of Surgery, Faculty of Medicine, Nursing & Health Sciences, Central Clinical School, Monash University, Melbourne, VIC 3800, Australia; philipmlewis@gmail.com; 5 Anatomics Pty. Ltd., Melbourne, VIC 3165, Australia; robert.thompson@anatomics.com

**Keywords:** 3D printing, additive manufacturing, implants, sintering, polymers, bone regeneration

## Abstract

The adoption of additive manufacturing (AM) techniques into the medical space has revolutionised tissue engineering. Depending upon the tissue type, specific AM approaches are capable of closely matching the physical and biological tissue attributes, to guide tissue regeneration. For hard tissue such as bone, powder bed fusion (PBF) techniques have significant potential, as they are capable of fabricating materials that can match the mechanical requirements necessary to maintain bone functionality and support regeneration. This review focuses on the PBF techniques that utilize laser sintering for creating scaffolds for bone tissue engineering (BTE) applications. Optimal scaffold requirements are explained, ranging from material biocompatibility and bioactivity, to generating specific architectures to recapitulate the porosity, interconnectivity, and mechanical properties of native human bone. The main objective of the review is to outline the most common materials processed using PBF in the context of BTE; initially outlining the most common polymers, including polyamide, polycaprolactone, polyethylene, and polyetheretherketone. Subsequent sections investigate the use of metals and ceramics in similar systems for BTE applications. The last section explores how composite materials can be used. Within each material section, the benefits and shortcomings are outlined, including their mechanical and biological performance, as well as associated printing parameters. The framework provided can be applied to the development of new, novel materials or laser-based approaches to ultimately generate bone tissue analogues or for guiding bone regeneration.

## 1. Introduction

Scaffolds for tissue engineering have historically been used as structures to support cell attachment, vascularisation, tissue growth, and regeneration [1,2]. Bone tissue engineered (BTE) scaffolds can mimic the role of native tissue, as substitutes [3], and/or encourage tissue ingrowth from surrounding bone [4]. The “ideal” BTE scaffold should also modulate cellular interactions, promote vascularisation, and replicate the mechanical properties at the target site [5,6]. Additionally, the preparation and sterilisation techniques need to comply with industry and regulatory standards [7]. To this end, this review explores the structure and function of bone, as well as the current strategies used to treat defects. It outlines the history of selective laser sintering (SLS) technology and how it can be utilised in BTE, including the use of SLS in the generation of scaffolds with defined porosities and interconnected pores that enable nutrient and waste diffusion. Each SLS material is discussion in the context of its chemical, physical, mechanical, and biological properties. Finally, this review examines the use of composite materials that more closely resemble native bone tissue by addressing some of the shortcomings associated with single-phase materials, including weak mechanical properties, lack of functionality, and their bioinert nature.

### 1.1. Bone Tissue Regeneration and Engineering

Bones provide structural integrity to the human body, protecting vital organs and facilitating mobility [8]. Bones are fundamental in maintaining homeostasis and play a role in energy metabolism [9]; thus, it is critical to preserve both their structural [10] and biological integrity [11]. Bone-related fractures directly cost the US economy USD 19 billion annually. The aging and growing population, along with a shift towards sedentary lifestyles, suggests this burden will undoubtedly continue [12]. Additionally, indirect costs such as loss of productivity and associated social implications highlight that bone-related diseases have significant health and economic repercussions [13].

Current treatments for bone defects resulting from congenital abnormalities, injury, or trauma typically utilise grafting. Bone grafting is a surgical procedure in which bone is replaced with other bone from the patient, a donor, or an animal. To date, the “gold standard” in bone grafting is autografting, where bone is harvested from non-essential bones, such as the iliac crest or mandibular symphysis, and used to replace defective bone. A shortcoming of autografts stems from the difficulty in preserving or obtaining specific geometrical features, particularly for maxillofacial reconstruction. Additionally, other issues arise due to donor site morbidity, increased risk of infection, haemorrhage, poor integration, nerve damage, and associated pain from multiple surgeries [14,15]. The most significant drawback with autografting arises when a significant amount of bone needs replacing and there is simply insufficient graft available within the same patient for transplant. Beyond autografting, exists allografting, where tissue is extracted from either living humans or cadavers and transplanted; xenografting, where tissue is transplanted across species, typically from bovine, porcine, or equine origins; alloplastic material grafting, where biomaterials are implanted as bone substitutes and composite grafts, including xenohybrids that combine synthetic biomaterials and xenograft bone grafts, as well as composites of multiple biomaterials, such as polymers, metals, and ceramics. Each technique is associated with shortcomings, ranging from broad ethical concerns, to immunological inadequacies risking tissue rejection [16,17].

Alloplastic materials, for instance, eliminate the need for a donor site; thus, limiting potential infections. They can be classified into nonporous, porous, and absorbable materials. Depending on the implant location, the type of material can vary. Typical alloplastic materials used in craniomaxillofacial applications are silicone, expanded polytetrafluoroethylene (Gore-Tex^®^) (Surgiform Technology, Lugoff, SC, USA), and High-Density Polyethylene Medpor^®^ (Stryker Corporate, Kalamazoo, MI, USA). Silicone is a nonporous material that prevents tissue infiltration, often leading to capsule formation and subsequent infection. Gore-Tex^®^ contains nodes and fibrils with a low porosity and a pore size ranging from 10–30 µm. Medpor^®^, made from high-density polyethylene, has a larger average pore size of 160–368 µm [18], although it lies in the lower range of porosity of human cancellous bone [19].

Common to many of the more successful bone graft substitutes is the integration of biomimicry into the scaffold design: closely replicating the natural composition, function, morphology, and mechanical properties of native bone to limit stress shielding, improve integration into surrounding tissue and, at times, instigate bone regeneration and remodelling proportional to implant degradation [2,3,13].

#### 1.1.1. Bone Structure and Repair

**Figure 1:**
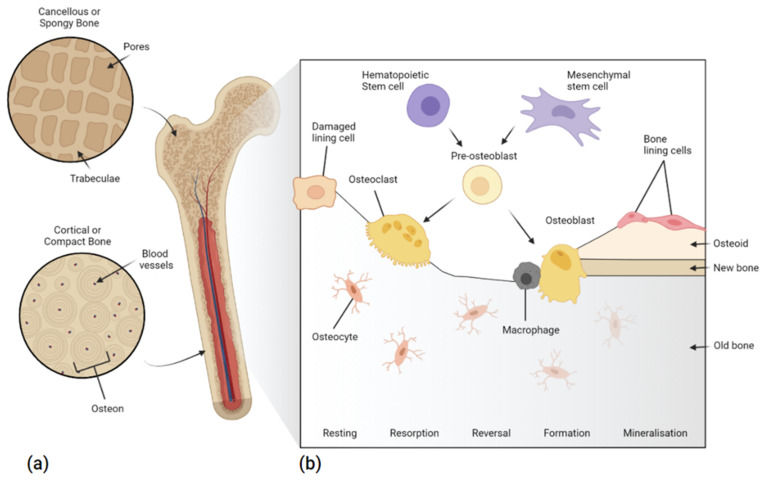
(**a**) Internal structure of human bone (**b**) bone remodelling and the stem cell pathway, including bone resorption by osteoclasts, bone formation, and mineralisation by osteoblasts, after which, osteoblasts become either new lining cells or mature into osteocytes.

The structure of bone is primarily made up of calcium crystals (hydroxyapatite~70 wt%) interspersed in a matrix of collagen among other mineralised extracellular matrices (ECM) and cellular components. There are two types of mature human skeletal bone, both of which are made up of osteons: cortical, and cancellous bone; sometimes also referred to as compact and spongy or trabecular bone, respectively [20]. The former is highly mineralised and dense, with a typical void porosity of approximately 10% and a range of 5–30%, resulting in a higher elastic modulus (17 GPa) [21] at the expense of toughness [22]. Cortical bone’s structure is made up of compact cylinders that serve to protect the inner cancellous bone. Cancellous bone is highly porous (30–90%), with a lower elastic modulus and tensile strength (<2 MPa) [23]. The irregular sponge-like structure [24], acts to absorb load, while creating a microenvironment for biological activity, surrounded by several organic components, including marrow, blood vessels, and cellular components (<2%) [22] (Figure 1).

With the exception of sesamoid bones, bones found in the human body can be categorised into long bones, short bones, flat bones, and irregular bones. Long bones, typically found in the arms and legs (humerus, femur, tibia etc.), are a hollow shaft or diaphysis made up of cortical bone and filled with bone marrow and adipose tissue. This is flanked by the epiphysis, which is made up of cancellous bone surrounded by a thin layer of cortical bone and acts to connect adjacent bones, to form joints. Short bones act to reinforce joints, while facilitating movement in areas such as the wrists and ankles (tarsals and carpals). They are made up of cancellous bone, surrounded by cortical bone. Likewise, flat bones are also made up of cancellous bone surrounded by cortical bone; although, flat bones’ primary function is to provide structure and protection, and thus are found in the cranium, scapula, sternum, ribs, and ilium. Irregular bones, found in facial regions and the spinal column, have complex geometries that aid in anchoring and protecting soft tissues, including providing an attachment point for the tongue and acting as a barrier for the spinal cord.

The ratio of cortical bone to cancellous bone varies depending on bone type and location. For instance, the cortical:cancellous ratio of vertebrae is 25:75, with the femoral head having 50:50, and radial diaphysis showing a 95:5 ratio. The quantity and proportion of cortical and cancellous bone at various sites affect the strength of bone independently. Additionally, most bone is anisotropic [25], where the response of the bone to a load depends on the direction of load application. For instance, longitudinally, bone is strong, yet when a load is applied to the surface of bone it is noticeably weaker. Thus, the “strength of bones are dependent upon the material, the microscopic structure and the shape of the whole bone” [26].

Bone is a dynamic tissue that undergoes continuous growth, modelling, and re-modelling, from foetal development until death. During development, bone growth occurs longitudinally along growth plates, where cartilage mineralisation forms primary new bone. Modelling occurs as a response to mechanical pressures, gradually adjusting bone structure in response to stimuli, in-line with Wolff’s law. This process does not involve the coupling of bone formation and resorption. Modelling occurs less frequently in adults than remodelling. Remodelling, on the other hand, is a continuous process that maintains bone mineral homeostasis with osteogenic cells. It includes the resorption of old bone tissue by osteoclasts, and the synthesis and mineralisation of the protein matrix by osteoblasts. In cortical bone, remodelling lasts approximately 120 days, while within cancellous bone, remodelling lasts 200 days [27]. Regulating these processes are osteocytes, which are terminally-differentiated osteoblasts that connect to and act as bone support structures at the bone surface (Figure 1). Bone metabolism is a continual process and is regulated by specialised cells and hormones, to preserve tissue strength and integrity, if compromised scaffolds can be used to restore balance.

#### 1.1.2. Scaffold Design

Similarly, to native bone, biomimetic bone scaffolds need to balance biological requirements with architectural intricacies and mechanical performance. Porosity, independently of material properties, is the percentage of void space in a solid. It is well established that highly porous (>75%) constructs, with interconnected pores, aid tissue ingrowth, by providing a large surface area for cells to attach and proliferate into, whilst enabling nutrient and waste transfer [28]. Increases in porosity, however, are inversely proportional to compressive strength [29], with a 10–20% porosity increase known to decrease strength up to four-fold [30,31]. Instead, other physical attributes, such as pore size, shape, and orientation, can be manipulated to improve biological outcomes while maintaining mechanical stability. Macropores larger than 100 µm [32] have been shown to promote osteogenesis and angiogenesis [32], whereas micropores smaller than 20 µm can stimulate mineralisation through improvements in cell recruitment and attachment [33,34]. The rate of tissue regeneration has been shown to be proportional to pore curvature, with concave pores observed to be better than flat or convex pores [35,36,37]. Fibroblasts have been shown to favour small pores over large pores. For example, beta-tricalcium phosphate (β-TCP) scaffolds with 100-µm pores and the lowest porosity (38%) showed improved bone ingrowth, both in vitro and in vivo, compared to scaffolds with 250 µm and 400 µm pore sizes and larger porosities [38]. In general, a distribution of pore shapes and sizes, and a high porosity that is well interconnected, without jeopardising the mechanical properties, will contribute to improved BTE scaffold outcomes.

The mechanical properties of implants should closely match those of the bone at the target site [39]. In load-bearing scenarios, discrepancies between bone and implant hardness can lead to stress shielding. Stress shielding is a phenomenon associated with implants that are harder or stiffer than bone (typically metallic implants), which prevent the mechanical load from being transferred to the surrounding bone tissue, provoking bone absorption. The absorption of bone from stress reduction gradually leads to bone resorption and the subsequent loosening of implants [40,41,42]. This loosening can lead to excessive oscillation of the implants, reducing osseointegration and causing chronic inflammation, pain, discomfort, and ultimately implant failure [43,44]. Thus, scaffold design should aim to incorporate materials with a similar Young’s modulus to native bone/tissue, to circumvent the possibility of stress shielding.

For rapid osseointegration, stimulating the interaction between implants and the surrounding tissue is imperative. Known as the bone–implant interface, this contact area has been shown to be the site of initial cell recruitment and adhesion. Thus, the surface characteristics down to the nanoscale can influence scaffold performance [45]. For instance, the surface roughness of metallic implants has been shown to be inversely proportional to biological fixation. Additionally, surface roughness has been shown to promote MSC proliferation, osteoblast differentiation, bone mineralisation, and growth factor production [46,47,48]. Although smoother surfaces have been shown to promote osteoblast spreading [49], at the expense of osseointegration [50], in practice, varying surface finishes can be productive in stimulating rapid cell attachment; although, challenges remain in optimising the application of these techniques. The use of computational stem cell proliferation and differentiation modelling may provide the necessary insights into understanding the interactions at the bone–implant interface [51,52]. Combining extensive practical approaches with computational models will aid in advancing the field of BTE.

#### 1.1.3. Regulatory Requirements for the Future of BTE Scaffolds

One of the most important requirements of any BTE scaffold or implant, apart from mechanical stability, is its biocompatibility. To ensure safety, medical device regulations prescribe requirements for demonstrating biocompatibility based on the intended use of the device, and which determine both the location and duration of implantation. Guidance on meeting these requirements are provided by regulators themselves; for example, in guidance documents published by the US Food and Drug Administration (FDA), with reference to standards such as ISO 10993: Biological evaluation of medical devices [53]. While, risk classification rules vary geographically, with the exception of most dental devices, permanent implants are classified as medium to high risk and fall within Class IIb/III according to the European Medical Devices Regulations (MDR) [54] and Class II/III by the FDA [55]. The risk classification then informs the required biocompatibility studies. For instance, permanently implantable medical devices require toxicological risk assessment of their chemical characteristics, as well as assessment of their nature, degree, frequency, and duration of contact with the body, with a range of in vitro and animal implantation studies. Device contact duration is categorised as limited (<24 h), prolonged (24 h to 30 days), or long-term (>30 days) [53,56]. Resorbable implantable devices have further specific requirements, to demonstrate that their degradation profiles are safe. Regarding 3D printed personalised implants, MDR classifies them as custom-made devices, where each individual device must be made using an MDR compliant technical file, typically under the control of an ISO13485 certified quality management system. In the USA, personalised 3D printed implants require preapproval via the 510(k) premarket notification pathway, with objective evidence proving substantial equivalence to an existing FDA-approved device with a similar risk profile and intended use case. Risk management of personalised implants can be categorised into the verification and validation of safety and performance, with reference to implant specifications and clinical use scenarios. The former, for instance, can use mechanical testing and finite-element analysis to determine implant compressive strength, as well as fixation requirements, from the length, thickness, quantity, and trajectory of screws. Surgically, cadaveric or model trial surgery can aid in implant validation, in-line with the requirements for traditionally manufactured implants. Compliance with regulatory requirements and international standards ensures a balance between innovation and safety, while also supporting the commercialisation and ultimately delivery of BTE scaffolds to patients [57].

#### 1.1.4. Advancing the Field of BTE

The shortcomings of the current grafting measures used for bone regeneration dictate that better, alternative approaches are needed, especially as these treatments will see increasing demand with our aging population. Tissue engineering approaches circumvent several of the shortcomings that arise from grafting, such as donor site morbidity and the ethical and immunological concerns associated with xenografting [58]. Among these approaches is the use of additive manufacturing technologies for BTE, which encompasses the added value of being able to manufacture patient-specific implants, to better fit and to better treat the patient. This review explores the use of a promising manufacturing technology, selective laser sintering (SLS), for producing robust, personalised tissue engineered alternatives. LS additive manufacturing approaches are highly versatile, enabling the fabrication of scaffolds from a range of biomaterials, including polymers, metals, ceramics, and composites; as outlined in the following sections. The limitations of the current methodologies and potential future strategies to circumvent hardware and material shortcomings are also discussed, to provide a future-facing perspective of the use of SLS in tissue engineering.

### 1.2. Laser Sintering Bone Tissue Engineering Scaffolds

The mid 1980s saw the development of additive manufacturing (AM), through the emergence of a technique termed stereolithography. AM technologies have been de-scribed as the “process of joining materials to make objects from three-dimensional (3D) model data, usually layer by layer, as opposed to subtractive manufacturing methodologies” by the ASTM International Committee F42. This form of manufacturing is considered part of the next industrial revolution [59]. AM techniques have more recently become known, more generically, as 3D printing. Historically, AM has primarily been used for rapid prototyping for research and development purposes [60] and has been shown to reduce development costs by up to 70% and time to market by 90%; both deemed to be vital in the development and delivery of patient-specific medical implants [61].

**Figure 2:**
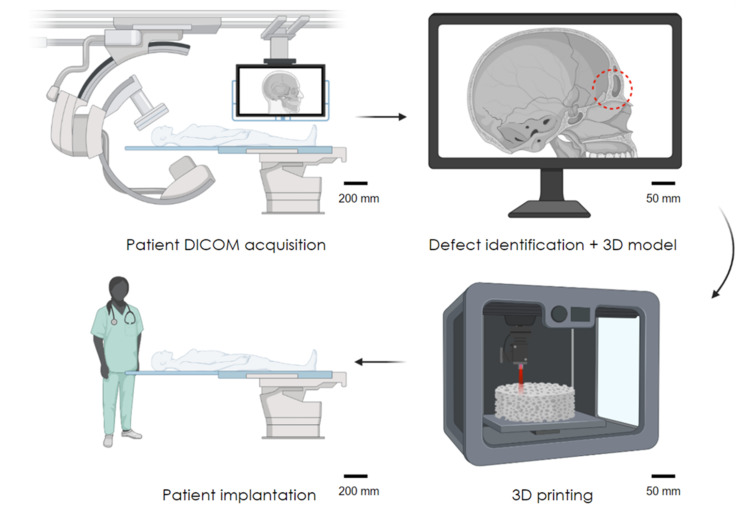
The process of 3D printing implants. Initial patient scans from X-ray, CT, or MRI to the development of a 3D model, following from patient scanning to modelling and implant generation.

The workflow for generating 3D printed medical implants starts with the patient. It involves the development of a 3D model through computer aided design (CAD) or reconstruction of 3D patient anatomical data from medical scans, such as computed tomography (CT/X-rays) or magnetic resonance imaging (MRI) [62], to create a series of 2D slice images, stored in the DICOM format (Figure 2). Once a 3D model has been generated and exported, most commonly as a Standard Tessellation Language file, it must be translated into machine readable code (typically g-code, which provides sequential machine movement instructions), which separates the model into distinct layers for printers to interpret and control the layer-by-layer fabrication process.

Three-dimensional printing is the layer-by-layer deposition of materials, to build a three-dimensional construct. Arguably the most common consumer 3D printing technique involves the controlled deposition of molten polymer, achieved by feeding a polymer filament (such as poly lactic acid) through a heated nozzle onto a platform where it solidifies [63]. Subsequent layers are fused on top of the previous layers, until a 3D object is realised. Other forms of 3D printing involve the use of lasers with curable liquid resins or powder beds. The former stereolithography involves a reservoir of photocurable resin, into which laser patterns are traced. Upon adhering to the print platform, subsequent solidified layers are added, until the construct emerges from the liquid resin. The latter, utilising a powder bed, termed powder bed fusion (PBF), has shown substantial promise as an AM technique capable of producing high strength constructs suitable as BTE scaffolds and are the focus of this review.

**Figure 3:**
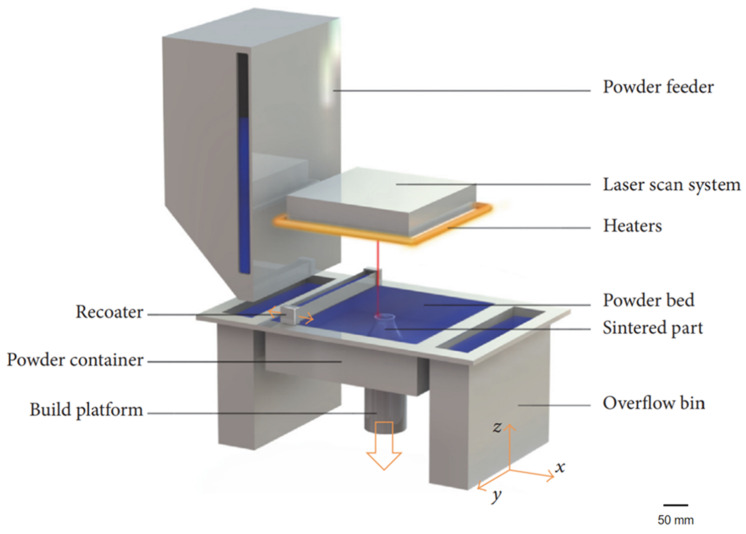
Schematic illustration of a typical laser sintering system. Scale bar = 50 mm. Reprinted with permission from [65].

In 1989, a master’s student at the University of Texas, Carl R. Deckard, designed, developed, and patented the first selective laser sintering (SLS) system. Following the initial development, the trio of inventors (Joseph J. Beaman and James F. Darrah) went on to create Nova Automation and DTM Corp, to industrialise and commercialise their technology. SLS is an AM approach that utilises PBF technology (Figure 3). In essence, both SLS and selective laser melting (SLM) techniques involve the localised heating or melting of a powder bed, with laser energy, which coalesces adjacent particles. For this to work efficiently, the powders must absorb the laser irradiation. Typically, both processes utilise infrared (IR) light lasers in a solid or gas state, as well as visible light diodes. Diodes drive solid-state lasers, where active ions of neodymium (Nd^3+^) are doped in a passive crystal of yttrium aluminium garnet; thus, producing a neodymium–yttrium aluminium garnet laser. These lasers can be guided by a fibre, to deliver 1064 nm light in concentrated areas for the laser melting of metals, such as stainless steel, titanium, and aluminium. Gas lasers lie deeper within the IR spectrum. At a wavelength of 10,600 nm, CO_2_ gas lasers are suitable for sintering polymers with high absorptivity, including polyamide and poly(ether ether ketone). More recently polyamide has also been processed through blue diodes at 445 nm; although for efficiency, the powder must be black or grey in colour. A detailed review exploring the types of lasers used in AM can be found here [64].

Though material absorptivity is a key component of sinterability [66], laser energy, exposure duration, laser spot size, scan spacing, layer thickness, and sintering temperature all play a vital role in effective SLS [67]. In both SLS and SLM, energy density is a fundamental factor that determines print quality. If the energy densities for sintering conditions are not optimised for the specific material used, the surface morphology [68] and porosity [69] may be poorly controlled, ultimately risking the production of fragile parts with dimensional inaccuracies [70,71]. To ensure consistency and fine resolution, the intrinsic and extrinsic material properties should be understood in the context of the chosen laser system, previously summarised in the context of polymer SLS [72]. There are five main polymer properties necessary to understand prior to exploring new or novel SLS powders. These properties stem from thermal, optical, and rheological factors, down to the material production and processing parameters; explored in depth later in this section.

To optimise the SLS process, an approximation of energy density can be calculated. Nelson, J.C. et al. [73] described the energy density (E) per unit area (J/mm^2^) of polymer-coated silicon carbide powders through the relationship of laser power (P) as a function of laser beam velocity (V) and scan spacing (S) (Equation (1)).

E = [P/(V × S)](1)

The equation was later amended to compensate for beam penetration and energy diffusion through a known volume [74], assuming the volume is optically transmissible, where T = layer thickness, given in (J/mm^2^) (Equation (2)) [75].

E = [P/(V × S × T)](2)

The model has limitations when modelling SLM, as the metals used radiate substantial energy through conduction [76]. Additionally, the simplicity of the equation cannot account for melt pool depth [77], nor keyhole porosities [78]; known phenomena in SLM [79]. The thermal conductivity of metal powders is important, and whilst it complicates SLM modelling, it has been shown to influence consolidation and part density, directly related to the bulk powder properties [80]. Assuming an evenly packed powder bed, the materials act as a heat transfer medium, capable of reducing thermal gradients and, thus, overcoming any deformation and warping. Likewise, the continual heating and cooling of the print environment has the same detrimental influence on part properties, including delamination, shrinkage, and warping, leading to morphological inaccuracies and potential mechanical instabilities [81]. Understanding the relationships between material and energy density will improve the print resolution, surface finish, and the overall mechanical properties.

## 2. Materials for Laser Sintering

**Figure 4:**
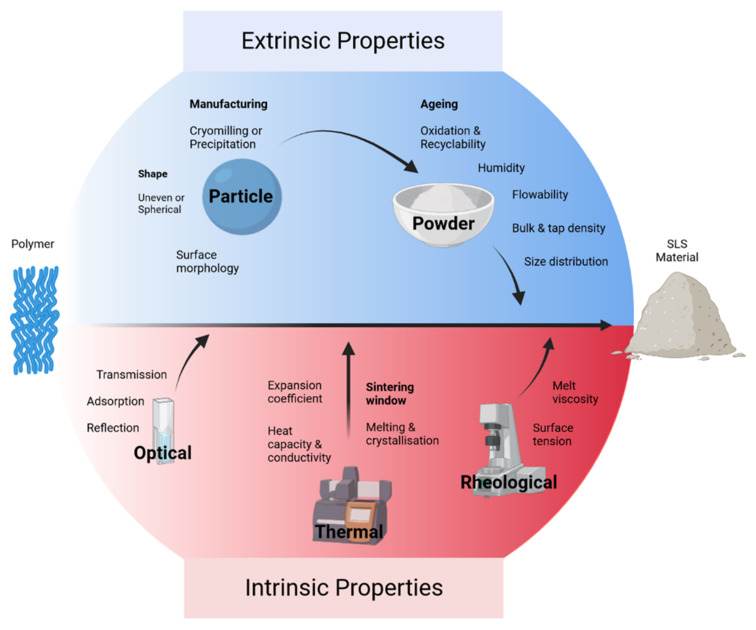
A summary of the extrinsic and intrinsic properties associated with the powder and process parameters that have an influence on materials used to produce parts via SLS.

There are many materials with the appropriate physical, chemical, and optical properties for SLS. These characteristics can be divided into extrinsic and intrinsic features (Figure 4). Apart from metals (in SLM systems) such as stainless steel, aluminium, and titanium; polyamides, polystyrene, polycarbonate, and ceramics are the most used materials in SLS systems [66]. However, polymers that can be made into a fine powder, also have the capacity to be sintered.

The most typical shape for particles is spherical, as the recoating blades or rollers can evenly distribute subsequent layers, due to their free-flowing capacity. Inconsistent particle size, aspect ratio, and shape, such as those generated via cryogenic milling, fail to yield dense parts and can result in weakened mechanical properties [82,83]. The size of particles for SLS systems range between 20 and 150 μm [84,85]; and for SLM, the most common range is between 20 and 60 μm [86]. Small particles are known to have strong electrostatic attractions, which can increase friction [87,88]; larger particle sizes, on the other hand, can reduce part finish and density [89]; thus, a range of particle sizes can be beneficial for improving flow and density. Additionally, environmental factors such as humidity also impact isothermal consolidation, but can be controlled with a shield gas such as Ar, N_2_, or He, among other inert gases.

**Figure 5:**
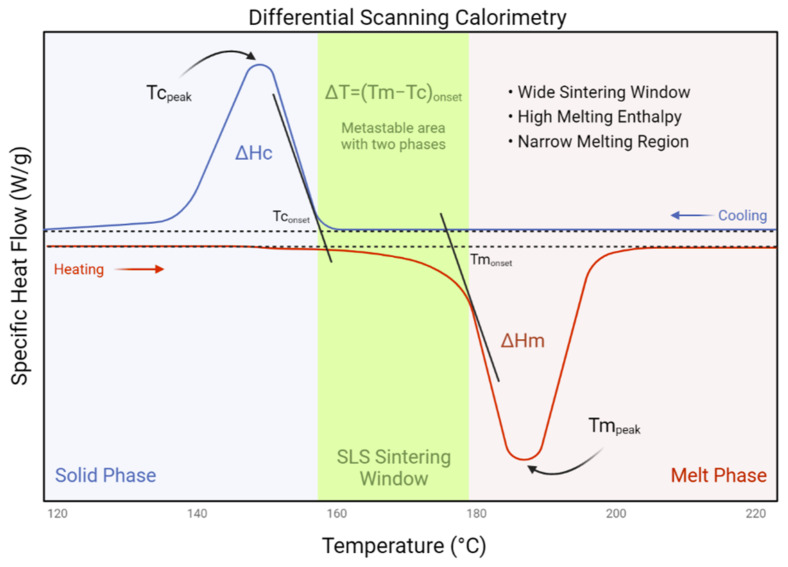
An ideal heat flow curve from differential scanning calorimetry analysis in the context of SLS printing, including a melt phase and solid phase determined from a typical heating and cooling rate of 10 °C/min. Adapted from [90].

Intrinsically, the thermal and optical properties need to be sufficiently understood for efficient particle fusion. Typically, semicrystalline polymers have suitable thermal characteristics to be processed through SLS. For new materials, a “sintering window” can be established through differential scanning calorimetry. Ideally, a distinct sintering window exists between the polymer melting point (Tm) and the crystallisation point (Tc). If the sintering window is narrow, printed constructs can deform or lateral growth can occur (Figure 5) [90]. Optically, many polymers contain aliphatic compounds (C–H) capable of absorbing portions of infrared radiation, particularly at the wavelength of 10–600 nm. Rheologically, appropriate SLS materials require a low melt viscosity and a low surface tension. A low melt viscosity is essential, as there is no compaction of polymer particles during the SLS process, when compared with injection moulding.

The ageing of polymer powders must also be considered when generating implants, with polyamide chain length being shown to grow with increasing build time and high build chamber temperature [91,92]. Due to the use of high energy lasers, the materials processed via SLS should undergo comprehensive physical and chemical analysis, as deformation [93] and chain scission [84] can be detrimental to scaffold and polymer stability. When compared to the bulk properties of the polymer, the tensile strength and modulus are comparable to sintered constructs; however, sintered parts are typically more brittle, with a reduced elongation at break [65,91].

### 2.1. Polymers

#### 2.1.1. Polyamide

Nylons are biocompatible polymers that belong to the family of polyamides (PAs) [94], and are used in a myriad of applications, extending from textiles [95] to biomedicine [96]. They are either derived from petroleum or natural sources such as castor oil [97]. Synthesis involves ring opening or condensation polymerisation. Nomenclature is based on the number of carbon atoms within each monomer, of which there are eight types. The most common commercially-available type of SLS is PA-12 (90–95% of the market), known under the trade names of PA 2200 (EOS, Krailling, Germany) and Duraform^®^ PA (3D Systems, Los Angeles, CA, USA) [98,99,100]. They are linear thermoplastics traditionally used in injection moulding. PA-12 has a broad processing window or “sintering window”, making it useful for SLS [101]. Additionally, it has a low melting viscosity and moisture absorption, superior elongation, wide range of melting and crystallisation temperatures, high flexibility and UV protection, when compared to the various other forms of nylon. However, PA-12 has a reduced elongation at break [102] and is more expensive when compared to other powdered polymers, particularly when producing suitable powder homogeneity for SLS [103]. This expense is due to the novel powder processing methods for making consistent powders for SLS; either by precipitation [104] or polymerisation [105].

Other forms of polyamides used in SLS are PA-6 and PA-66, as they are known to have a molecular structure resembling that of the collagen found in human bone [106,107]. On a larger scale, biomimetic architectures of trabecular human bone can extend outside the achievable resolution of typical LS systems (≤50 μm). A previous study rectified this issue by scaling up bone CT/MRI data four-fold to generate PA-6 scaffolds mimicking human bone, while broadening the porosity and interconnectivity requirements for adequate bone regeneration [108]. Printed scaffolds were tested both in vitro and in vivo with porcine bone marrow stromal cells and in a porcine mandible, respectively. Bone tissue infiltration after 6 weeks was 43.2%, compared to the 50.3–65% observed following implantation of HA scaffolds with the same pore geometry [109]. The reduction in tissue growth was thought to be associated with material leaching due to partial sintering. Additionally, the bioinert nature of PA could have played a role in reducing the tissue ingrowth. Similarly, PA was used in a recent study that employed an SLS technique to overcome stress shielding by generating porous, biomimetic trabecular-like bone scaffolds [110]. To address this, a porous honeycomb structure was generated through SLS using PA-66. The elastic modulus of scaffolds was found to be within the range of trabecular human bone (50–500 MPa). The mechanical data were then applied to a finite element simulation, to predict how changes in porosity (between 59 and 96%) influence elastic modulus. A nonlinear relationship was found between an increase in elastic modulus and decreased porosity. This model could also be applied to a patient’s CT data, to predict the mechanical properties of bone at a defect site, to avoid stress shielding.

#### 2.1.2. Polycaprolactone

Polycaprolactone (PCL) has been widely used in BTE strategies. PCL is produced by polymerisation of ε-caprolactone via cationic, anionic, or radical polymerisation methods [111,112]. It is a biodegradable [113,114], semicrystalline, aliphatic thermoplastic with a glass transition temperature (Tg) of −60 °C [115], and with a typical melting point of ~60 °C [116,117,118], tuneable down to 46 °C [119]. The availability of different molecular weight PCL’s results in tailorable degradation kinetics over months or years, depending on the tissue engineering application [117]. Its low cost and favourable physico-chemical attributes make it suitable for a wide range of AM techniques, including FDM [120], melt electrowriting [121], and SLS applications.

**Figure 6:**
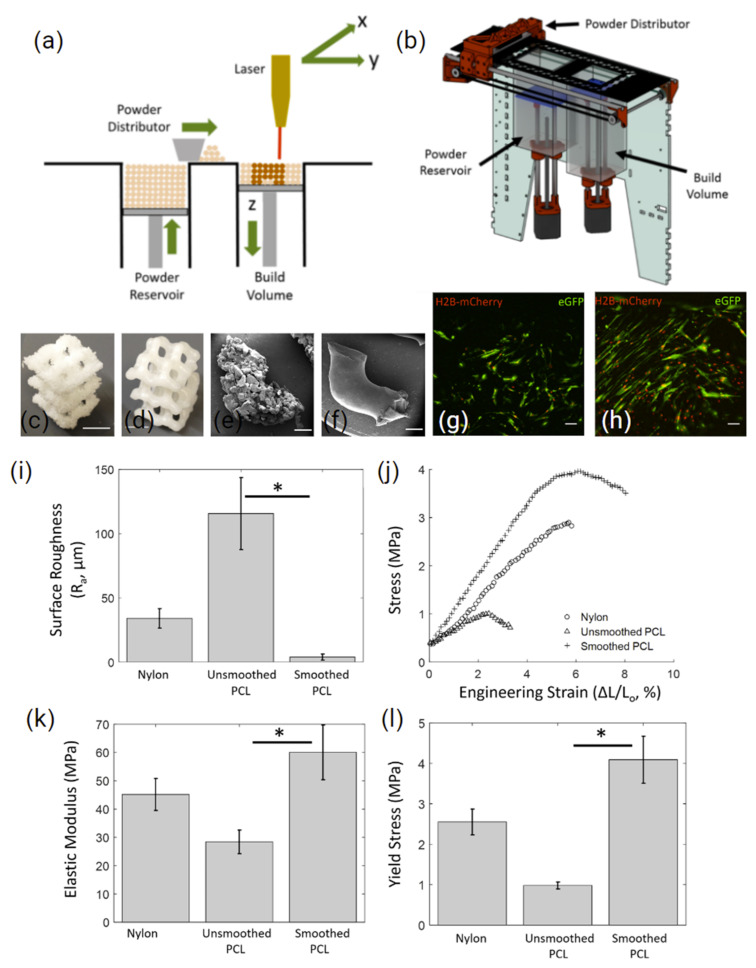
(**a**,**b**) A schematic and 3D render of the custom recoating platform developed to build up PA 12 and PCL powder in the Z direction with the use of a laser cutter. Surface finish of PCL sintered diamond lattice, before (**c,e**) and after vapour smoothing (**d**,**f**) (scale bars = 1 mm), (**g**) shows hMSC morphology on sintered PCL and (**h**) shows hMSC morphology on vapour-smoothed sintered PCL (scale bar = 1000 μm). (**i**–**l**) Surface roughness and mechanical properties of sintered PA 12 and PCL as well as vapour-smoothed sintered PCL. * denotes *p* < 0.01 using Student’s *t*-test. Plots represent mean ± SD. Adapted from [122].

A novel study by Kinstlinger et al. [122] explored the interfacing of a custom recoating platform (Figure 6a,b) with a laser cutter to process PA-12 and PCL. They were able to reproduce sophisticated lattice structures recapitulating bone structures (Figure 6c,d). Additionally, the study explored the influence of the post-processing of SLS prints on mechanical properties and studied the biocompatibility of the structures using human MSCs (hMSCs). Following 5 min of vapour smoothing with dichloromethane, scaffold surface roughness was significantly reduced, while the elastic modulus and yield stress were improved (Figure 6k,l). Following 10 days of in vitro cell culture, the seeded hMSCs showed minimal morphological change (Figure 6g) compared to the elongation and spindle-like morphology observed on the vapor-smoothed surface (Figure 6h). A similar study by Mazzoli, et al. [123] utilised a Sinterstation CO_2_ laser system to fabricate PCL discs (15 mm diameter) with 500 µm pores. The print parameters used included a bed temperature of 50 °C, a laser power of 12 W, and a 0.1 mm layer thickness. This resulted in a compressive strength of 3.6 MPa at 48% porosity, the lower range of trabecular bone [124]. Additionally, seeded hMSC demonstrated spherical and branched morphology, confirming biocompatibility in vitro.

More recently, Gu et al. [125] utilised small (50 µm) and large (150 µm) PCL microspheres to create bilayered cartilage and subchondral bone scaffolds. They compared three different structures: non-channel, consecutive-channel, and inconsecutive-channel. A biomimetic hierarchical structure with varying channels was designed, to prevent vascularisation on the dense surface, while the porous phase beneath was used to promote osteogenesis and vascularisation. The dense, non-channel scaffold had a compressive strength of 18.27 MPa, with the consecutive-channel and inconsecutive-channel resulting in 5.91 and 10.26 MPa, respectively. The native osteochondral tissue of rabbits was measured to be 20–30 MPa [126]. In vitro all scaffolds supported MSC adhesion, proliferation, and spreading. Interestingly, in vivo, the inconsecutive-channel scaffold showed a significantly higher bone volume fraction and trabecular number. This was in contrast to the non-channel scaffolds, which showed limited tissue integration, with the consecutive-channels revealing inconsistent tissue ingrowth. Overall, this hybrid SLS printing approach demonstrates a novel way of tuning the mechanical and biological properties of scaffolds without the need for cell or growth factor loading; expanding the potential of PCL in BTE applications.

#### 2.1.3. Polyethylene

Polyethylene (PE) is a thermoplastic polymer of ethylene with a variable crystalline structure. PEs are produced at almost 5 million tonnes per annum [127], making it the world’s most common plastic. They have a broad range of applications, due to their ease of production, ranging from packaging [128] to biomedicine [129]. PE is classified by its branching and density, and exists as ultra-high, high, medium, and low molecular weight varieties. Each form of PE varies in its mechanical, chemical, thermal, optical, and electrical properties, broadening its applicability. Due to its long-term stability and biocompatibility [130], PE is one of the most used materials for alloplastic surgical implants and has been used in hundreds of products, ranging from facial implants [131], through to coatings for oesophageal stents [132], as well as in total hip arthroplasty [133].

SLS of PE is challenging, due to its narrow sintering window, which can impact the printing accuracy [134]. Without fine tuning the energy density, laser energy can broadly radiate into surrounding particles, leading to lateral growth and warping; in turn, filling voids [135] and reducing part porosity [136]. Additionally, in its native state, PE is white or semi-transparent, making it highly reflective to visible (445 nm) or near infrared (1064 nm) light. Using CO_2_ lasers (10,600 nm), however, polyethylene appears opaque, improving the sintering potential. Additionally, the porosity of the printed part can be tuned when printing with a CO_2_ laser [137], which can be beneficial for BTE applications.

Another component capable of influencing porosity is the size of the powder particles. Samoria et al. [138] investigated pore size as a function of HDPE powder size, using commercially available HDPE particles with size ranges of 106–125 µm, 150–212 µm, and 212–380 µm, respectively, and were able to control pore gradients. A larger particle size yielded significantly more closed pores, at the expense of mechanical strength, when compared to smaller particles. They concluded that the discrepancies between mechanical properties were a result of limited necking of adjacent particles. A more recent study compared commercially available porous HDPE implants with SLS printed scaffolds in vivo [139]. They found that the SLS printed scaffolds demonstrated higher scaffold porosity compared to traditional moulding, and this supported good tissue integration after implantation. Additionally, the functionalisation of the HDPE surface using plasma was also demonstrated to improve the formation of blood vessels within the implant, enabling more rapid tissue ingrowth and maturity [139]. Overall, although PE has been used sparingly in SLS systems, due the limitations mentioned, it has established uses in biomedicine, warranting further exploration within the BTE and AM landscapes.

#### 2.1.4. Polyetheretherketone

Polyetheretherketone (PEEK) is a semi-crystalline polymer that is stiff, robust, and lightweight [140], with decades of use in the aerospace, medical, and dental fields. It has exceptional strength, a Young’s modulus of 3.6 GPa and tensile strength of 90–100 MPa [141], a high wear resistance and low friction coefficient, rendering it favourable as a biomaterial, to mimic the native properties of bone. With a typical glass transition temperature of 143 °C, a melting point of 343 °C [142], and thermal degradation at 575 °C [143], it can sustain high temperature exposure for extended periods. Its high melting point inspired the modification of conventional SLS systems [144], leading to the birth of high temperature (>300 °C) SLS or HT-LS [145,146].

One study explored the generation of patient-specific PEEK cranial implants via SLS. The study compared print orientations and found that vertically-printed SLS constructs were not as accurate or strong as horizontally-printed SLS constructs, with a 70% reduced failure rate [147]. Interestingly, when compared to injection moulding, SLS-generated PEEK scaffolds showed a reduced tensile strength but an improved compressive strength.

However, the high temperature processing parameters for PEEK sintering limit reusability. This heightened sensitivity stems from PEEK’s cold powder coating preparation, which can lead to crystallisation shrinkage and warping deformation if the powder bed temperature fluctuates [148]. The high temperatures and print duration impact the physico-chemical properties of the polymer, severely reducing PEEK’s reusability, even when using an inert gas shield [149,150]. These changes caused by ageing, can reduce powder performance, resulting in inconsistencies between printed scaffolds, even with the same printing parameters.

To improve the bone–implant interface, intricate internal architectures have been generated via SLS from PEEK [151]. By mimicking trabecular bone and impregnating constructs with a co-culture of ADSCs and BMSCs, scaffolds improved cell morphologies, resembling that of fibroblasts and leading to greater osteogenic differentiation of the ADSCs. Overall, SLS fabricated porous PEEK, combined with novel co-culturing techniques is a promising approach for generating patient-specific craniofacial implants.

### 2.2. Metals

**Figure 7:**
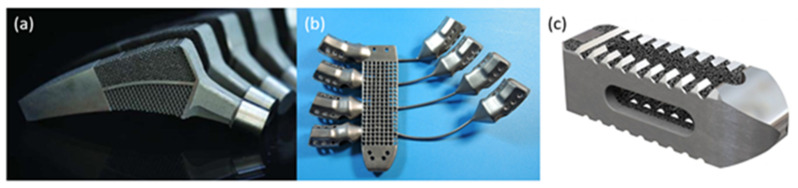
Examples of metallic AM implants using EBM and SLM processes, specifically (**a**) titanium alloy femoral stem implant with complex lattice structures for improved Osseo integration by TU Delft using SLM, (**b**) patient-specific titanium sternum and ribs using EBM by Anatomics, (**c**) serial produced titanium posterior lumbar cage with porous structures using SLM by Stryker [159].

AM has matured from a rapid prototyping tool to a serial production technology capable of reliably producing end-use metallic parts for the medical and aerospace industries. In particular, PBF metallic AM techniques such as SLM and electron beam melting (EBM) are currently suitable methods for producing high-strength medical devices, for load-bearing applications such as orthopaedics, and for aerospace components, as both industries rely heavily on strict manufacturing protocols and associated quality assurance, to ensure safe and reliable products. SLM and EBM can construct highly dense components with a good surface finish and mechanical properties exceeding the equivalent wrought or cast metallic parts [152]. The design freedom available to engineers through AM allows the manufacture of complex-shaped components optimised for specific applications, such as meta-biomaterial lattice structures for enhanced osseointegration and patient-specific or serial-produced orthopaedic implants, as shown in Figure 7.

In load-bearing applications, metal implants can lead to stress shielding, a loosening at the bone–implant interface, which can, in turn, lead to infections [153,154]. Lattice structures, with designed or pre-set unit cell configurations are used to reduce the bulk stiffness and weight of a metallic implant, while promoting cell adhesion, to maximise osseointegration and minimising aseptic loosening [155]. Likewise, porosity can also impact stiffness and anisotropy, while influencing permeability [156] and cell infiltration [157]. These features can be designed with the aid of finite element analysis, to generate biomimetic structures more closely resembling bone itself [158]. An extensive review by Tan et al. [86] explored the use of PBF for manufacturing metallic scaffolds, with a particular focus on biocompatibility, topology, and mechanical properties for orthopaedic implants.

Current research trends are moving towards a meta-biomaterial and biomimetic approach to enhancing patient-specific implants, using different design approaches. The complex geometry achievable with AM allows for the development of new meta-materials with properties difficult to find in nature, such as a negative Poisson ratio, negative compressibility, and negative stiffness, coupled with high specific properties [155]. These properties may have potential applications for specific implant scenarios, such as the examples shown in Figure 7a–c, where a combination of negative and positive Poisson’s ratios was used to allow the hip implant to expand into the hip bone under the tensile and compressive loads induced by bending, to eliminate debonding at the implant–bone interface [159].

### 2.3. Ceramics

Historically, ceramics have played a significant role in the development of bio-mimetic bone tissue engineering strategies [160]. Specifically, calcium phosphate (CP)-based ceramics closely mimic the inorganic phase of the bone matrix, both in structure and chemical properties, and are typically fabricated into porous biomimetic scaffolding, to recapitulate the native bone matrix [161]. Examples of common ceramics include hydroxyapatite (HA, Ca_10_(PO_4_)_6_(OH)_2_), β tricalcium phosphate (βTCP, Ca_3_(PO_4_)_2_), and calcium carbonate (CC, CaCO_3_). These materials have long-standing clinical histories in a range of bone cements [162], including Pro-Osteon^®^ (HA and CC) [163], Norian CRS (CP) [164] and Vitoss (CP) [165], as well as in surface coatings and slow-release drug delivery systems [166]. Additionally, a range of bioactive glasses (BG), most notably the FDA approved Bioglass 45S5 formulation, as developed by Hench [167], have also shown substantial promise for bone tissue engineering; given their comparable mechanical properties to the ceramic phase of native bone, combined with surface properties conducive to osteoblast adhesion and proliferation [168].

SLS/M strategies for the fabrication of ceramics pose a number of key challenges in both processing and post-processing, which have limited their widespread application in bone tissue engineering research [169]. Ceramics, in general, exhibit very high melting temperatures, typically between 1000 °C and 1800 °C; therefore, they require high-powered lasers capable of heating ceramic powders to above these temperatures to selectively densify ceramic powder particles into 2D layers. Ceramics are, therefore, highly susceptible to sensitivities in inter-layer binding; and high heating and cooling rates, coupled with the poor intrinsic thermal shock resistance of ceramics, can lead to thermal stress-induced crack formation and brittleness [169]. In addition, the use of biological additives, including growth factors or drugs, which may be beneficial in bone tissue engineering strategies, cannot be concurrently processed [170].

**Figure 8:**
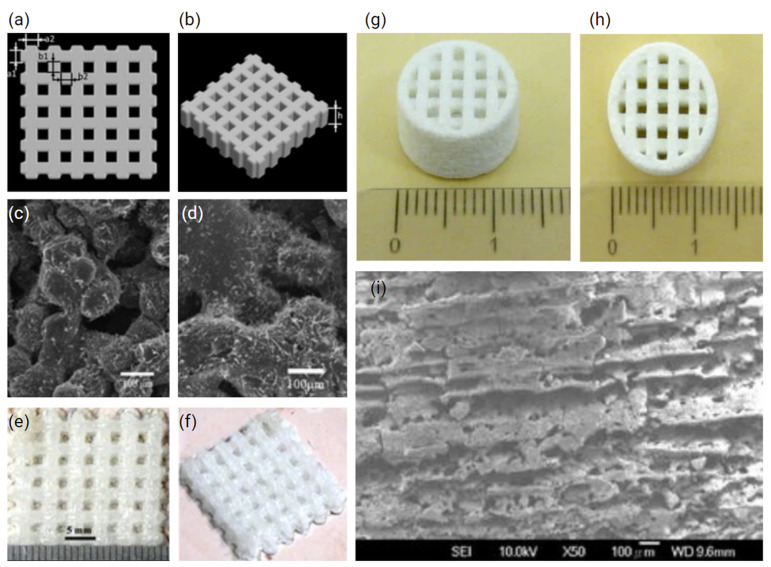
(**a**,**b**) CAD of crosshatch scaffolds using SolidWorks^®^ (version 2011, Dassault Systèmes SolidWorks Corporation, Waltham, MA, USA). (**c**,**d**) Scanning electron micrographs and (**e**,**f**) overviews of SLS 45S5 Bioglass scaffolds fabricated via SLS. Adapted from [172] (**g**–**i**) HA-nanosilica sol composite scaffolds. Adapted from [173].

Despite these challenges, recent studies have reported the fabrication of mechanically robust βTCP [171] as well as 45S5 Bioglass scaffolds, the composition of which was strategically transformed into Na_2_Ca_2_Si_3_O_9_, a favourable crystallisation phase, through optimised heating and cooling during SLS (Figure 8a–f) [172]. The feasibility of fabrication of HA and nanosilica sol composite scaffolds via SLS has also been demonstrated, using a custom-made SLS printer (Figure 8g–i) [173]. Here, scaffolds with 750 to 1050 µm pores were produced, with the assistance of a post processing heat treatment at 1200 °C, to further bind the SLS layers and improve mechanical stability. Characterisation of surface roughness using atomic force microscopy (AFM) also identified the effectiveness of producing 525-nm rough scaffolds using SLS, which were conducive for the attachment of bone marrow-derived osteoprogenitor cells [173].

The processing of ceramics via SLS-based approaches has been challenging, given the thermal and mechanical properties of ceramics [174,175]. Optimisation of the binding properties of LS ceramic scaffolds has been a major focus of research for strategies applied to BTE, since native bone tissue exhibits elastic properties not typically characteristic of brittle ceramics [176]. Several strategies have been widely explored to overcome the aforementioned challenges [177]. These include heat treatment post-processing [178,179] and the use of binding materials [180] and reinforcing additives, such as metals [181,182] and other ceramics [183], for multiphasic properties. Most favourably, composites with thermoplastics, metals, and other more-readily processable materials have been extensively investigated, which will be discussed in more detail in the following section.

### 2.4. Composites

The primarily homogenous materials explored, so far, have met the necessary print requirements for LS applications. However, bone itself is a heterogeneous composite material and is perhaps nature’s best example of a composite structure which requires different phases for optimal form and function. To satisfy its osteogenic capacity, bio-mimetic scaffolds for BTE need to comply with both the physical and biological attributes of native human bone. Polymers alone are known to be relatively bioinert and mechanically weaker than cortical bone; however, they are very easy to form into different shapes and sizes. Ceramics and metals can often be too hard and brittle, resulting in stress shielding or implant instability. One solution involves the use of composite blends of materials, to exploit the favourable properties from each, while reducing their negative attributes [4]. For instance, the mechanical strength of polymers can be improved through fibre and ceramic reinforcement [176], and their bioinert nature can be enhanced via the addition of bioactive compounds to promote osteogenesis [85].

**Figure 9:**
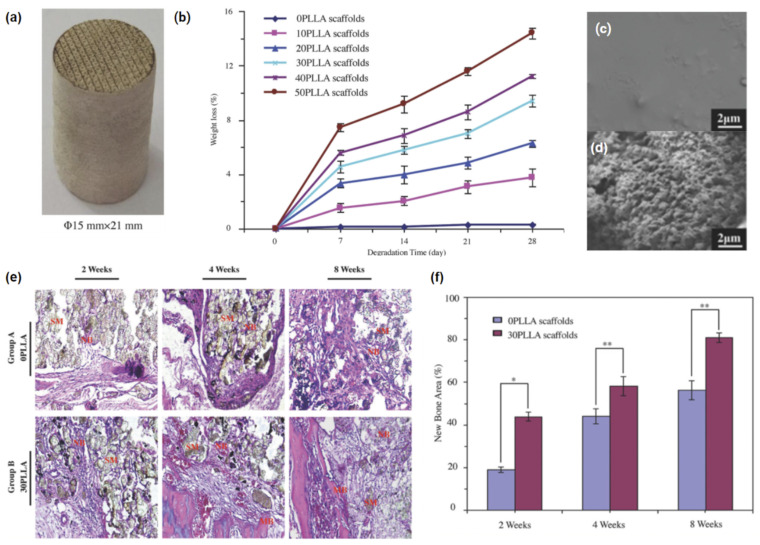
(**a**) Sintered composite cylinder containing PEEK/β-TCP/PLLA (5:2:3 wt/wt/wt), generated with Solidworks (version 2011, Dassault Systèmes SolidWorks Corporation, Waltham, MA, USA) and converted to stereolithography (STL) format prior to printing with a CO_2_ laser system (Rofin-Sinar Laser GmbH, Hamburg, Germany). A spot size of 500 µm, scan velocity of 120 mm/s, interval of 950 µm, and a layer thickness ranging from 0.1–0.2 mm were used. (**b**) Weight loss behaviour of the scaffolds during a 28 day PBS immersion, where the number represents the weight percentage of PLLA. (**c**,**d**) SEM micrographs of constructs with 0 and 30 wt% PLLA after 28 days in SBF solution. Histological images and quantitative analysis of new bone formation. (**e**) H&E staining images of the bone defect sections in the experimental group A and experimental group B after 2, 4, and 8 weeks of surgery (SM: scaffold material; NB: new bone; MB: mature bone). (**f**) Quantitative analysis of new bone (* *p* < 0.05, ** *p* < 0.01). Adapted from [184].

One of the first instances of composite sintering without organic solvents was described in 2003, where Tan et al. [71] physically blended HA (10–40 wt%) and PEEK powders, to form various grades of HAPEEK. Through increased laser energy they were able to SLS the composite at much lower part bed temperatures when compared to other studies of HT-LS. Unfortunately, with an increased HA ratio, the constructs became fragile and brittle, suggesting that high HA composition may not be suitable for load bearing implants. On the other hand, when HA particles were embedded into a polymer phase and partially exposed, this was potentially beneficial for improving the PEEK’s long term mechanical properties and osteogenic capacity. Fent et al. [184] incorporated a biodegradable polymer with a bioactive PEEK composite containing β-tricalcium phosphate (β-TCP), the three-phase material was SLS into porous cylinders (Figure 9a) with a CO_2_ laser and a broad 500-µm spot size. The degradation rate was able to be tuned over 28 days, by adjusting the concentration of the polymer Poly-l-lactic acid (PLLA) (Figure 9b); thus, improving the appetite forming capacity of the scaffolds, due to an increased surface area and exposure to integrated β-TCP (Figure 9c,d). Following an 8-week rabbit implantation, H&E staining reiterated the improved bone forming ability (Figure 9e,f).

Polyamide (PA 12) was blended with HA (78 wt%) to overcome the modulus mismatching common with homogenous materials [185]. The study found that part porosity was significantly influenced by the thickness of the deposited powder layers, and more so than laser energy density. HA nanoparticles have also been introduced to coat and reinforce PA 12 [186], revealing a 15–20% improved tensile strength and modulus, at the expense of reduced elongation at break. Likewise, porous PA-HA composites have been sintered in various orientations, to assess the impact on mechanical properties, both practically and theoretically [187]. Interestingly, vertically sintered dog bones had an improved compressive and tensile strength when compared to those horizontally sintered, while strength improved overall with increased HA concentration, from a 10.6 MPa tensile strength with PA-HA 95%:5% to 24.3 MPa in the PA-HA 80%:20%; similar trends were also observed following compression tests. PA has also been combined with glass beads [188] and carbon nanofibres [189], to improve the storage modulus by 22% and tensile modulus by 1000 MPa, respectively. The former, however, found the morphology of cryo-processed powder to be undesirable for SLS, and this resulted in uncontrollable surface morphologies.

HA has also been integrated into other polymers for SLS. HAPEXTM is a polyethylene composite containing 40% Vol of bioactive synthetic hydroxyapatite filler [190]. As a bone analogue, HAPEXTM aims to overcome stress shielding and bone resorption at fixation points. The reinforcement of HDPE with HA improved the fracture toughness, over the purely ceramic material, whilst retaining its osteoconductivity in vivo [191]. A study by Savalani et al. [192] explored the use of 30 and 40% HAPEXTM in SLS systems. They compared the use of both CO_2_ and Nd:YAG lasers for powder coalescence. Interestingly, the “sintering window” of the CO_2_ laser was determined to be wider; thus, consistent printability was achievable, though fine optimisations were necessary. Slower CO_2_ laser scanning velocities (below 600 mm/s at both 3.6 W and 7.2 W) resulted in polymer degradation, as the energy density was too high; on the other hand, at a scan velocity of 4800 mm/s and 7.2 W laser energy, the SLS parts become too brittle from insufficient necking/coalescence; thus, a 1200 mm/s scan velocity was deemed suitable. For a comprehensive review of AM approaches using HA composites, the reader is referred to a recent study by Milazzo et al. [193].

Composite SLS approaches have been used to improve osteogenesis. One study explored the use of PCL/HA microspheres in SLS. Specifically, they created multi-layered constructs that ranged from pure PCL to PCL + 30 wt% HA nanoparticles in 5 wt% increments throughout seven 400 µm layers [194]. The biomimetic gradient construct, from top to bottom, was intended to replicate articular cartilage and subchondral bone, respectively. Following a 12-week implantation in a rabbit model, gradient constructs showed improved trabecular bone formation when compared to pure PCL scaffolds, after µCT analysis. This was also consistent with both immunohistochemical staining for both cartilage and bone-specific proteins and the upregulation of chondrogenic and osteogenic genes following qRT-PCR analysis. Protein expression for both aggrecan (AGG) and collagen type II (COL II) were observed to be stronger in gradient scaffolds compared to the PCL and untreated control groups. On a genetic level, the relative mRNA expression of chondrogenic markers AGG and COL II, as well as osteogenic markers collagen type 1 and osteocalcin, were all significantly upregulated in the gradient constructs. Another study utilising a PCL/HA composite created using SLS compared a range of nano-HA concentrations (i.e., pure PCL, PCL with 5 wt% nano-HA, PCL with 10 wt% nano-HA, and PCL with 15 wt% nano-HA). Interestingly, using the same SLS parameters, scaffold porosity reduced, and the compressive strength improved with increased nano-HA concentration. With pure PCL resulting in an approximately 78.5% porosity and 1.38 MPa compressive strength, and PCL with 15 wt% nano-HA showing a porosity of 70% and a compressive strength of 3.17 MPa. Following a 9-week rabbit femur implantation, the highest concentration of HA resulted in enhanced bone formation [195]. Another study explored the use of biodegradable polymer microspheres with an osteoconductive element. The polymer matrix was made up of either PHBV or PLLA and the bioactive element was either Ca-P or CHAp. The incorporation of calcium phosphate nanoparticles, improved SaOS-2 proliferation and ALP expression over the virgin PLLA scaffolds [85]. Several other papers have explored the use of HA reinforced composites for BTE (Table 1).

## 3. Conclusions and Future Directions

The incidence of bone related diseases and injuries is growing with the global ageing population. AM strategies currently hold significant promise for addressing many of the shortcomings associated with traditional bone grafting methods to treat these conditions, from constructing patient-specific implants directly from medical scan data, to generating intricate internal architectures that recapitulate the hierarchical structure and dynamic mechanical properties of bone. Furthermore, the ability to create composite materials through a combination of bioactive materials with structurally robust or elastic materials, whilst adding various cell types and biological cues, enables a toolbox of options tailored to specific tissue types.

As the AM technologies develop, so does our understanding of the relationship between print method, materials, and the human body. Further understandings, both qualitatively and quantitatively, between material and laser interactions may provide insights into the use of new materials for laser sintering. Additionally, new mechanisms for feeding material into the print bed may broaden material applicability. For instance, digital light processing systems have established rotating bed recoating systems to deposit microparticle layers of HA and TCP, to produce high-resolution parts for bone tissue engineering applications. Similarly, layer-wise slurry deposition has been developed to improve print bed powder density during printing, and thus improve the mechanical properties of prints. Combining these novel deposition techniques in laser sintering systems could hold the key to producing biomimetic bone for generating patient-specific implants.

Promising new advancements such as EBM [205] for metal printing to the use of bioactive coatings [204], antimicrobials, and even drug delivery methods for PBF [206,207,208,209,210,211], will ensure that novel implants can be provided to patients in a timely manner, with the appropriate legislation and oversight from government and regulators. In creating these personalized implants, humans will be equipped with the necessary tools to mitigate the impact of bone-related illnesses and the overall disease burden. This burden can lead to superfluous stress on the healthcare sector and unpredictable economic impacts. These new AM approaches also have the capacity to broaden the accessibility of the technology in the developing world, so that one day we can provide objective patient care and potentially engineer patient-specific tissues on a global scale.

## Figures and Tables

**Table 1:** Summary of composite sintering approaches outlining the specific print parameters utilised, physical attributes, and biological outcomes of the printed constructs. Where P = Laser Power, λ = Wavelength, S = Scan Spacing, T = Layer thickness, V = Scan Velocity, Φ = Beam Diameter, E = Elastic Modulus, σUC = Ultimate Compressive Strength.

Composite Formulation(s)	Print Specifications	Physical Attributes	Biological Response	Ref.
PCL/HA In wt% ratios of 100:0, 90:10, 80:20 and 70:30	P = 1–1.2 W λ = 10.6 µm S = 152.4 µm T = N/A V = 914 mm/s Φ = 450 µm 50 °C bed temp	Increased HA concentration resulted in a higher E but a reduction in σUC	-	[196]
PCL/β-TCP In wt% ratios of 100:0, 90:10, 50:50, NB 50:50 utilised smaller PCL particles	P = 7 W λ = 10.6 µm S = N/A T = 0.11 mm V = N/A Φ = 410 µm 49 °C bed temp	Increasing β-TCP content was found to decrease the strength	In vivo bone formation significantly lower in PCL/TCP sintered composite compared to pure β-TCP	[197]
PLLA/GO@Si-HA	P = 3.5 W λ = N/A S = N/A T = N/A V = 180 mm/s	Compressive strength and modulus improved by 85% and 120% after incorporating GO@Si-HA, with a marginal improvement in hardness	4 wk SBF: PLLA minimal, PLLA/GO minimal, PLLA/GO@Si-HA significantly improved appetite formation and MG-63 cell morphology and ALP activity after 7 days	[198]
PEEK PEEK/20%plyglycolicacid (PGA) PEEK/40%PGA	P = 100 W (max) λ = 10.6 µm S = 2.5 mm T = 0.1–0.2 mm V = 400 mm/min Φ = 800 µm	Increase in PGA concentration reduced compressive and tensile strength	PGA had no significant influence on MG-63 cell viability or morphology	[199]
Poly (vinylidene fluoride)/Bioactive glass 58s (PVDF/58s)	P = 100 W (max) λ = 10.6 µm S = 3 mm T = 0.1–0.2 mm V = 500 mm/min Φ = 800 µm	BG was found to be slightly exposed on the surface of scaffolds following EDS analysis	BG 58s addition improved osteoconductivity and osteoinductivity of scaffolds, following SBF and MG-63 cell seeding analysis	[200]
Aliphatic-polycarbonate/HA(a-PC/HA) a-PC a-PC/5 wt% HA a-PC/10 wt% HA a-PC/15 wt% HA	P = 11 W λ = 10.6 µm S = 0.15 mm T = 0.15 mm V = 2000 mm/s Φ = 200 µm 135 °C bed temp	Surface roughness and porosity (53 to 82%) increased with HA content, below 15 wt% ideal 6–7 times reduction in scaffold strength with HA compared to pure a-PC	Osteoconductivity unchanged by SLS processing	[201]
Poly[3,6-dimethyl-1,4-dioxane-2,5-dione]/HA	P = 10 W λ = 1.06 µm S = N/A T = N/A V = mm/s Φ = 125 µm	Young’s modulus increased from 6.4 to 8.4 GPa with HA addition	Sintered composite scaffolds improved ATSC attachment and viability, compared to foaming method and virgin polymer	[202]
PVA/HA 90:10 vol% 10–75 µm 50–100 µm	P = 10–20 W λ = 10.6 µm S = N/A T = N/A V = 1270–2540 mm/s & 2032 mm/s 65–75 °C bed temp & 80 °C bed temp for larger particles	Ball mixing was found to be best for homogenous blends of PVA and HA when compared to tumbler mixer. Larger particles also prevented clumping during layer deposition	-	[203]
PCL PCL/TCP PCL/TCP/collagen	P = 1 W (PCL) & 2 W (PCL/TCP) λ = N/A S = 0.2 mm T = N/A V = 500 mm/s 40 °C bed temp	Significant improvement of compressive modulus with addition of TCP, col no difference	Improved pASC attachment, viability and osteogenic differentiation (ALP and osteocalcin) with TCP and TCP/col addition, ALP activity highest at day 7 for all scaffolds (over 28 days). Woven bone and vasculature observed in vivo with composites, pure PCL was full of fibroblasts and granular tissue	[204]

## Data Availability

The data presented in this study are available on request from the corresponding author.
